# Categorical Vowel Perception Enhances the Effectiveness and Generalization of Auditory Feedback in Human-Machine-Interfaces

**DOI:** 10.1371/journal.pone.0059860

**Published:** 2013-03-19

**Authors:** Eric Larson, Howard P. Terry, Margaux M. Canevari, Cara E. Stepp

**Affiliations:** 1 Institute for Learning and Brain Sciences, University of Washington, Seattle, Washington, United States of America; 2 Department of Speech, Language, and Hearing Sciences, Boston University, Boston, Massachusetts, United States of America; 3 Department of Biomedical Engineering, Boston University, Boston, Massachusetts, United States of America; University of South Florida, United States of America

## Abstract

Human-machine interface (HMI) designs offer the possibility of improving quality of life for patient populations as well as augmenting normal user function. Despite pragmatic benefits, utilizing auditory feedback for HMI control remains underutilized, in part due to observed limitations in effectiveness. The goal of this study was to determine the extent to which categorical speech perception could be used to improve an auditory HMI. Using surface electromyography, 24 healthy speakers of American English participated in 4 sessions to learn to control an HMI using auditory feedback (provided via vowel synthesis). Participants trained on 3 targets in sessions 1–3 and were tested on 3 novel targets in session 4. An “established categories with text cues” group of eight participants were trained and tested on auditory targets corresponding to standard American English vowels using auditory and text target cues. An “established categories without text cues” group of eight participants were trained and tested on the same targets using only auditory cuing of target vowel identity. A “new categories” group of eight participants were trained and tested on targets that corresponded to vowel-like sounds not part of American English. Analyses of user performance revealed significant effects of session and group (established categories groups and the new categories group), and a trend for an interaction between session and group. Results suggest that auditory feedback can be effectively used for HMI operation when paired with established categorical (native vowel) targets with an unambiguous cue.

## Introduction

Human-machine interfaces (HMIs) are designed to translate volitionally produced physiological signals into commands or control signals to augment or restore normal user function. For example, a common goal of HMI designs is to improve communication or mobility for patients with spinal cord injury, stroke or amyotrophic lateral sclerosis (ALS), in whom typical motor function has been greatly reduced or eliminated. HMI designs typically utilize biosignals such as electro-encephalography (EEG) or surface electromyography (sEMG) (which translate scalp potentials or muscle activities, respectively, into control signals). In many HMI designs, users are required to imagine specific motor movements [1] or fixate on a target in a visual scene to evoke P300 [2] or steady-state visual responses [3]. Although effective, these types of HMI designs rely on visual feedback or sustained visual attention for operation, thereby limiting normal user visual function. In an attempt to overcome this, multiple auditory-based HMI designs have exploited the cortical potentials evoked by presented auditory stimuli with increasing success [4]–[7].

Unfortunately, recent studies on HMI designs utilizing the auditory modality have found that auditory feedback is inferior to visual feedback, both in terms of resulting participant performance [8] and required participant training time [9]. Nonetheless, it has been shown that audio-visual training of spectrally-complex auditory categories (via implicit association with visual stimulus categories) could be used to obtain accurate participant categorization of novel tokens from the trained auditory categorical distributions [10], suggesting that audio-visual feedback can be used to train auditory categorization.

Utilizing auditory categorical perception is complicated by the fact that participants can experience greater difficulty forming multi-dimensional auditory categorical judgments than unidimensional judgments [11]. However, auditory categorization of vowel sounds requires multi-dimensional categorization in the formant one – formant two (F1–F2) plane and listeners are able to quickly categorize these sounds for speech perception. Critically, listeners tend to perform effective discrimination only among those vowel categories that are perceptually relevant during early language acquisition [12]. Moreover, these learned vowel categories exhibit a so-called perceptual magnet effect, whereby similar repeated stimuli both become more easily categorized yet less readily discriminable [13]. For example, listeners perceive novel vowel categories – those not utilized in their native language – in terms of the perceptual categories of their native language [14], even when explicitly trained to learn the new perceptual categories [15], and listeners only distinguish between sounds belonging to one particular category when explicitly trained to do so [16]. It is not surprising that recent a recent study utilizing motor imagery and implanted cortical electrodes [17] showed high performance by mapping two dimensional control to two dimensional vowel (formant) space.

Here we aim to determine the specific effects of underlying native two-dimensional vowel categorization abilities of listeners on HMI control in order to inform future development of effective auditory HMIs. To obtain a high signal-to-noise ratio (SNR) control signal to test the effectiveness of our auditory vowel-production feedback, here we utilize sEMG as it provides signals several orders of magnitude larger in amplitude than EEG. The sEMG-based system utilized here provides real-time feedback to participants while requiring only a USB-based soundcard and sEMG amplifier connected to a standard laptop running custom C++ software (see Methods), but other control signals (e.g., EEG) could be substituted in principle. After training participants to produce specific vowel sounds based on continuous auditory and visual feedback, we found that participants readily transferred ability to control the HMI using auditory feedback alone. Critically, we trained two thirds of these participants using vowel categories from their native language (American English), with half of them receiving both auditory and text (e.g., an example word) vowel cues and the other half receiving only auditory vowel cues; the remaining third were trained on non-standard vowel categories spanning the same formant plane and containing similar frequency content (cued with auditory stimuli). Although all participants could be trained to achieve trained vowel targets, the participants operating on standard English vowel categories proved to be more efficient in generalizing their training to produce vowel sounds from novel categories (either standard or non-standard vowel categories) only when explicitly reminded of the vowel using visual cues. This suggests that utilizing clearly cued native vowel category percepts (and exploiting the corresponding perceptual magnet effect) enhances the effectiveness of the HMI and generalization to novel vowel outputs.

## Methods

### Participants

**Figure 1:**
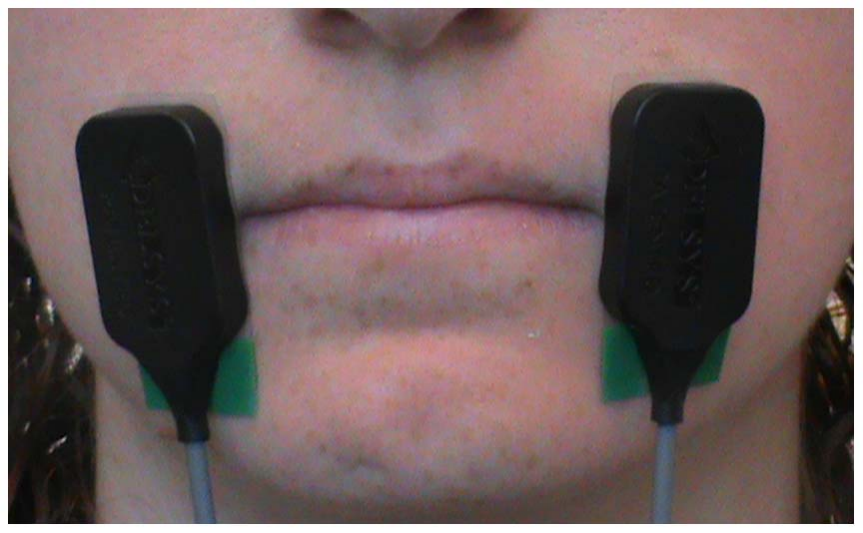
The sEMG signal used to control human-machine-interface operation was obtained from electrodes placed on the left and right orbicularis oris muscles of each participant, who then underwent training phases to become accustomed to controlling the synthesized vowel production caused by activating the left and right muscles.

This study was approved by the Boston University Institutional Review Board. All participants completed written consent. The individual in Figure 1 has given written informed consent (as outlined in PLOS consent form) to publish this photo. No work took place outside of the authors' country of residence (United States of America). Twenty-four native-English speaking participants were randomly chosen to be in one of three possible groups: those receiving “established categories” via either auditory and text cuing or auditory cuing, and those receiving “new categories” via auditory cuing. In the established categories group with auditory and text cues (EC-AT), there were 5 females (mean age = 22.4 years, SD = 3.4 years) and 3 males (mean age = 21.3 years, SD = 2.1 years). In the established categories group with only auditory cues (EC-A), there were 3 females (mean age = 21.3 years, SD = 3.2 years) and 5 males (mean age = 20.2 years, SD = 1.3 years). The new categories (NC) group consisted of 6 females (mean age = 23.2 years, SD = 3.3 years) and 2 males (mean age = 21.0, SD = 0.0 years). No participants reported having any speech, hearing or neurological disorders.

### Equipment Set-Up

In order to control the two-dimensional formant movement in the F1–F2 plane, we measured the activation of the orbicularis oris muscles using a sEMG system (Delsys 2-channel Bagnoli System) in combination with an external sound card (M-Audio Fast Track PRO). The sEMG system contained a bandpass filter of 20–450 Hz. The bilateral orbicularis muscles were chosen to provide high signal-to-noise ratios for two-dimensional control, although in principle other muscles or sources of control could be used. Double differential electrodes were placed on both the left and right orbicularis oris muscles (see Figure 1). Before placing the electrodes, the skin was prepared by cleaning with alcohol and peeling (exfoliation) with tape. Once the sEMG electrodes were attached to the skin using double-sided adhesive interfaces, medical-grade tape was used to further secure the electrode placement. Both electrodes fed into the sEMG system, and the two channels were amplified by a gain of 1000. The ground electrode (Dermatrode) was placed on the center of the forehead. The signals from the sEMG channels were fed into the M-Audio external sound card and then on into the computer. Auditory feedback was provided via a loudspeaker placed in front of the participant.

### Software Set-Up

Custom C++ software translated measured sEMG signals into formant production and provided auditory and visual feedback. In the software, a viewing window consisted of a two-dimensional space in which the x-axis corresponded to F1 values, and the y-axis corresponded to F2 values. F1 values were limited between 300 Hz–1.2 kHz and were controlled by activation of the right orbicularis oris muscle; F2 values were limited between 300 Hz–4 kHz and were controlled by activation of the left orbicularis oris muscle. The sEMG power ranges were normalized on a per-participant basis. The sEMG signals were recorded while participants were instructed to cycle between periods of rest and maximum voluntary contraction (MVC). A MATLAB (Mathworks, Inc.) calibration script determined the participants' MVC as well as noise floor. The participant's maximum and minimum power (S_max_ and S_min_) for each channel (right/F1 and left/F2) were then used to map activity onto formant locations. The maximal formant value was achieved when the power was S_max_ –0.15 (S_max_ – S_min_), and the minimal formant value was achieved when the power was S_min_ +0.10 (S_max_ – S_min_), with a linear mapping between formant locations and activations in between. The resulting range of sEMG values employed (10–85% MVC) balanced the required level of sensitivity (dynamic range) with the prevention of user discomfort and fatigue due to near-maximal contractions.

### Experimental Paradigm

Each group (EC-V, EC-A, and NC) participated in four sessions of 120 trials over three days, each session lasting approximately 40 minutes. The first and second session (training) were completed on days 1 and 2, respectively. On the third day, the participants completed both session 3 (training) and 4 (generalization). Auditory feedback and cues were always produced by using a Klatt synthesizer [18] as implemented in the STK toolkit [19] using a fundamental frequency of 125 Hz; visual feedback and text cues varied based on the session and group as detailed below.

**Figure 2:**
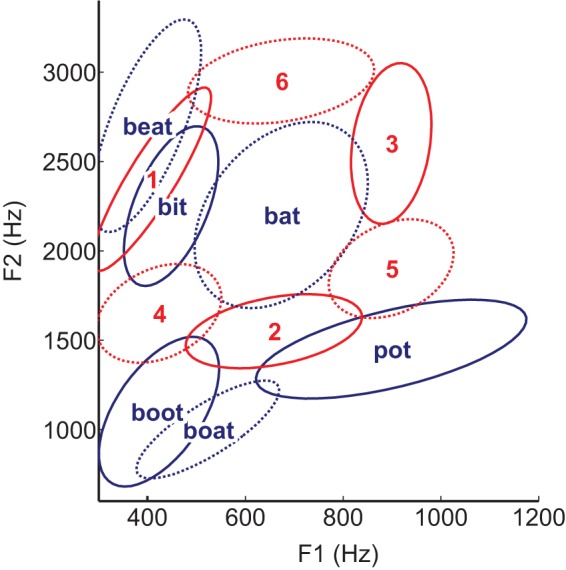
Categorical (red) and non-categorical (dark blue) vowel targets are shown by their designated ellipses in the formant (F1–F2) plane. Categorical vowels were assigned exemplar words that were shown in the center of the ellipse or center of the screen for the established categories text cue (EC-AT) group, depending on the session, to cue the participant which vowel sound to produce. Solid ellipses designate the vowel targets trained and tested in the first three sessions, while the dashed ellipses designate novel (untrained) vowel targets participants had to generalize to in the final (fourth) session.

For the EC-AT group, participants were instructed to learn to use muscular contraction to reach a target vowel sound in the two-dimensional space. Participants always received their target in two forms before each trial: an auditory cue of the vowel sound as well as the presentation of a fixed sample word that contained the sound (text cues). The three training targets used for sessions 1, 2, and 3 were fixed as ellipses in the F1-F2 plane associated with the American English vowels/I/,/u/, and/α/; these targets used the cue words “bit”, “boot”, and “pot”, respectively. For session 4 (generalization), the novel targets were fixed as ellipses in the F1-F2 plane associated with the American English vowels/i/,/æ/, and/o/with the cue words “beat”, “bat”, and “boat”, respectively. At the beginning of each trial in every session, the target vowel sound was presented with the both the auditory cue and the visual text cue (the sample word). Across all sessions, subjects received real-time auditory feedback as to their location in the F1-F2 plane. However, the visual feedback that the participants received in order to find their target varied depending on the session (see Table 1 for a depiction of these differences, described in detail below). In all sessions the participant's goal was to modulate the sEMG activation to cause the production of a particular vowel in the F1–F2 plane, represented by an ellipse that covered the acceptable range of formant one and formant two values for that particular vowel sound. See Figure 2 for an illustration of vowel targets. These target ellipse locations were determined based on American English production data [20]. Participants had 15 seconds to reach the bounds of the correct vowel ellipse (i.e., produce the correct vowel sound).

**Table 1:** The four sessions for the two different training groups (categorical and non-categorical) used different levels of audio-visual feedback.

Participant Group	Session	Real-time visual feedback	Ellipse	Text cue	Real-time auditory feedback	Auditory cue
Established categories visual cues	1: Auditory & full visual	✓	✓	On Ellipse	✓	✓
	2: Auditory & partial vis.		✓	On Ellipse	✓	✓
	3: Auditory only			Centered	✓	✓
	4: Auditory only (novel)			Centered	✓	✓
	Refresher	✓	✓	On Ellipse	✓	✓
Established categories auditory cues	1: Auditory & full visual	✓	✓	None	✓	✓
	2: Auditory & partial vis.		✓	None	✓	✓
	3: Auditory only			None	✓	✓
	4: Auditory only (novel)			None	✓	✓
	Refresher	✓	✓	None	✓	✓
Non-categorical	1: Auditory & full visual	✓	✓	None	✓	✓
	2: Auditory & partial vis.		✓	None	✓	✓
	3: Auditory only			None	✓	✓
	4: Auditory only (novel)			None	✓	✓
	Refresher	✓	✓	None	✓	✓

In session 1, participants in the EC-AT group received real-time visual feedback in the form of a gray dot that moved across F1–F2 plane based on the sEMG signal. Participants were also shown the target ellipse location in the two-dimensional space, with the text cue appearing at the center of the ellipse during the trial. To indicate that the participant had correctly localized the target ellipse (holding the dot within it), the ellipse became a darker color. Participants were also told that once they became used to manipulating the sEMG to reach the targets, they should begin focusing on using the auditory real-time feedback in order to locate the target. This provided participants with continuous audio-visual feedback training. For session 2, the real-time visual position feedback (gray dot) was removed. The target ellipse was again shown with the text cue centered on it, real-time auditory feedback was present, and the target ellipse in the F1–F2 plane darkened when participants were within the target ellipse. Participants were again encouraged to continue focusing on using the continuous auditory feedback in order to find the target. This provided participants with a continuous audio and discrete visual feedback training. For session 3, target ellipses were never shown. The auditory cue and text cue were presented at the beginning of the trial to designate the target, but the text cue was positioned in the center of the screen so participants could only use auditory information to infer the location of the target. Since no feedback was provided regarding whether or not they were at the target (other than the trial ending after one second of holding the correct position), this session provided only continuous auditory feedback. Session 4 was identical to session 3, except that the participants were instead tested on three novel targets (vowel sounds/i/,/ae/, and/o/) rather than the training targets. Participants could only use the real-time auditory feedback to find the target vowel, again presented to them using an auditory cue and the centered text cue, requiring them to perform auditory generalization. Before session 2 and session 3, 15 trials with audio-visual feedback (each equivalent to a single trial from Session 1) were completed to re-orient participants to using the sEMG.

Training and testing of the EC-A group was the same as in the EC-AT, except they were never given text cues for the target vowel sound. In other words, in session 1, they received real-time visual feedback in the form of a gray dot and were shown the target ellipse location in the two-dimensional space, but no text cue appeared at the center of the ellipse during the trial. Likewise, no text cue was shown for sessions 2–4. Nonetheless, as in the EC-AT group, an auditory cue was always provided before the start of the trial, alongside a visual ellipse target location for sessions 1–2 (and no visual cue for sessions 3 and 4).

Training and testing of the NC group was the same as EC-A, except that these participants received a fixed set of three training targets consisting of vowel-like sounds not present in American English in sessions 1–3 and a different fixed set of novel targets for generalization consisting of three vowel-like sounds also not present in American English for session 4. These vowel-like sounds were similar in frequency content to vowel targets presented to categorical participants, but with loci in the F1–F2 plane not associated with American English vowels. See Figure 2 for an illustration of vowel-like targets for the EC-AT, EC-A, and NC groups throughout each session. Participants in the NC group were also not presented with text cues to designate which particular sound they would be hearing (since there were no example words in American English corresponding to the target vowel sounds).

### Analysis

We measured performance (percentage of targets reached by a user within a session) and reaction time (time to target) for each subject. To assess differences across participant groups (EC-AT versus EC-A versus NC) and training sessions (1–4), we used a two-way (one-within [session], one-between [group] participants) analysis of variance (ANOVA), employing a conservative Greenhouse-Geisser non-sphericity correction. *Post-hoc* tests were administered using paired (or un-paired for across-group comparisons) *t*-tests with a stringent Bonferroni correction; all reported p-values are corrected.

## Results

**Figure 3:**
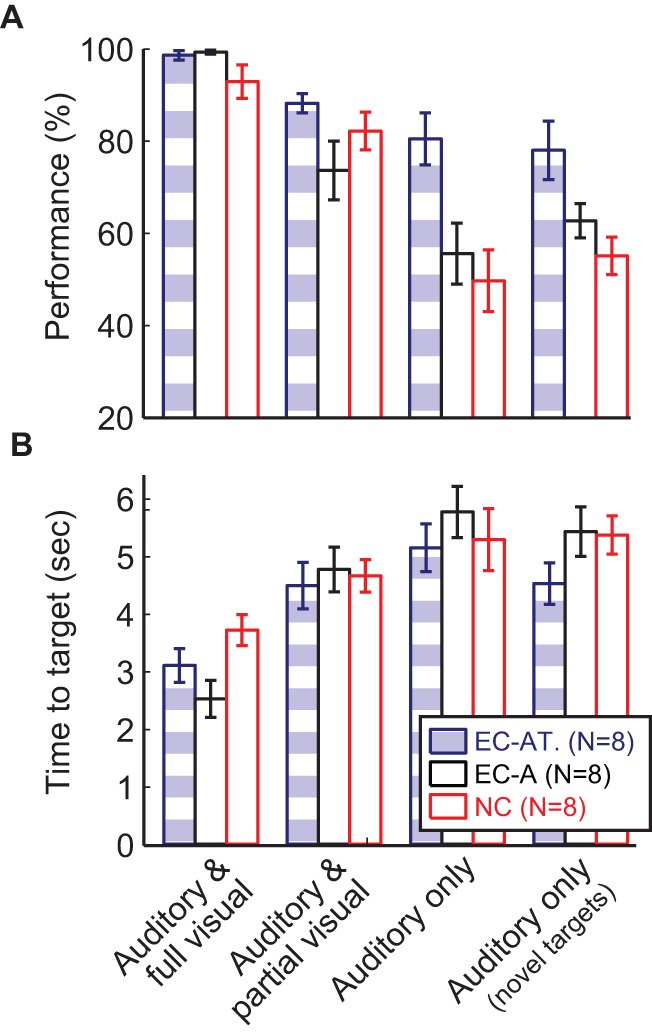
Participant performance in terms of percent of performance (targets reached; A) and time to target (reaction time; B) are shown for established categories with auditory and text cues (blue and white striped; EC–AT), established categories with only auditory cues (black; EC–A), and new categories (red; NC). While the time-to-target data did not show differences between training schemes, the percent correct performance showed significant differences across training groups: the EC-AT group performance was significantly higher than the EC-A and NC groups (both *p_adj_* <0.002). There was a trend for an interaction between session and training group, suggesting that categorical vowel perception aided participants in utilizing and generalizing auditory feedback (third and fourth sessions). Error bars show ± 1 standard error.

We compared HMI control of participants in the three training groups in each of the four behavioral sessions: those with 1) auditory and full visual feedback applied to training targets; 2) auditory and partial (discontinuous) visual feedback applied to training targets; 3) auditory feedback only applied to training targets; and 4) auditory feedback only with novel (untrained) vowel targets (see Figure 3). Using a two-way ANOVA (see methods and Table 2), we found a significant main effect of session (*p*<0.001) and training group (established categories using text cues [EC-AT] versus established categories without text cues [EC-A] versus new categories [NC]; *p*<0.001), as well as a trend for an interaction between session and training group (*p* = 0.055) on performance (see Figure 3A). The time-to-target (i.e. reaction time) measurements (Figure 3B; two-way ANOVA) were generally noisier, but nonetheless revealed a significant main effect of session (*p*<0.001) with no significant effect of training group (*p* = 0.427) or interaction between session and group (*p* = 0.203). Using post-hoc paired t-tests to examine performance and reaction time across sessions, the first session (auditory and full visual feedback for training targets) was significantly better than sessions 2–4 (all *p_adj_* <0.001), and session 2 (auditory and partial visual feedback for training targets) was significantly better than sessions 3 (auditory feedback only for training targets) and 4 (auditory feedback only generalized to novel targets), with all *p_adj_* <0.006 for performance, all *p_adj_* <0.038 for reaction time); no significant difference between sessions 3 and 4 was found for performance or reaction time (*p_adj_* ≥ 0.05). Using post-hoc unpaired t-tests to examine overall performance differences between groups, the EC-AT group was significantly better than the EC-A and NC groups (both *p_adj_* <0.002), while the EC-A and NC groups were not statistically significantly different (*p_adj_* >0.5).

**Table 2:** Two-way ANOVA results for performance (percent of targets reached by each participant in a session) and reaction time (RT).

Performance: Within-Subjects Effects						
Source	Sum of Squares	Df	Mean Square	F	Sig.	η_p_^2^
Session	18737.3	2.2	8661.5	35.0	<0.001	0.63
Session * Group	2623.1	4.3	606.3	2.5	0.055	0.19
Error (Session)	11229.7	45.4	247.2			
**Between-Subjects Effects**						
Group	4898.1	2.0	2449.0	13.8	<0.001	0.57
Error	3709.1	21.0	176.6			
**RT: Within-Subjects Effects**						
Session	74.1	2.4	29.8	26.5	<0.001	0.56
Session * Group	8.5	4.9	1.7	1.5	0.203	0.13
Error (Session)	58.8	52.3	1.1			
**Between-Subjects Effects**						
Group	3.2	2.0	1.6	0.886	0.427	0.08
Error	38.8	21	1.8			

To determine specific performance differences that led to the trend for an interaction between behavioral session and training group, we used *post-hoc* unpaired t-tests across training groups within each session. The differences between the EC-AT and NC training groups for sessions 1–4 were *p_adj_* >0.5, *p_adj_* >0.5, *p_adj_*  = 0.036, and *p_adj_*  = 0.11, respectively. Differences between the EC-AT and EC-A groups were *p_adj_* >0.5, *p_adj_* >0.5, *p_adj_*  = 0.16, and *p_adj_* >0.5, respectively. Finally, differences between the EC-A and NC groups were all *p_adj_* >0.5. Even employing the conservative multiple-comparisons adjustments (Bonferroni correction), session 3 performance for EC-AT remains significantly higher than for NC while the difference in session 4 is trending toward significance. This suggests that participants in the EC-AT group performed significantly better than participants in the NC group when using auditory only feedback during trained targets (session 3), as well as possibly when generalizing to novel targets (session 4). Moreover, the trend for an interaction between session and group appears driven by the enhanced performance of the EC-AT group compared to the NC group.

## Discussion

Our study focused on HMI-based control of continuous vowel synthesis based on a high-SNR input (sEMG) utilizing audio and visual feedback. In line with previous studies on the implementation of auditory feedback in HMI designs [8]–[10], [21], we found that participant performance in operating a HMI utilizing auditory feedback alone is close to (but slightly less than) that utilizing (audio-)visual feedback. However, while previous work has shown users have difficulty forming new two-dimensional categorical judgments of auditory stimuli [11], we find that utilizing a user's existing categorical vowel perception allows for two-dimensional categorization and HMI control based on auditory feedback alone that is comparable to full audiovisual feedback. Critically, this performance increase was dependent upon the presentation of the target using a visual word cue; when an auditory vowel cue (consisting of a synthesized vowel sound) was used, performance decreased.

### Effects of Categorical Perception

In *categorical perception* distinct perceptual categories are mapped onto physical quantities that vary continuously. This phenomenon is seen in a variety of percepts in vision (e.g., color [22] and facial expressions [23]) and notably in speech perception [24]. Although the formants produced by altering the vocal tract shape during voicing can be varied continuously, listeners perceive these continuous changes in spectral peaks as distinct productions of learned vowel categories [13]. Similarly, these learned vowel categories display the perceptual magnet effect such that stimuli within the category are more easily categorized and less discriminable [13]. Although previous studies have shown that users show low performance in integrating auditory feedback for motor control in HMIs [8], [9], here we show that leveraging the perceptual expansion of certain physical stimuli by using known perceptual categories as motor targets can drastically improve performance as evidenced by the very large effect size (η_p_^2^  = 0.6). Specifically, in session 3 the EC-AT group performance was 30.8% greater than the NC group, a ∼60% increase; in session 4 the EC-AT group was 22.9% better, a ∼40% increase. This significantly improved performance of the EC-AT group compared to the NC group in the auditory-only and auditory-generalization tasks suggests that previously established categorical vowel perception enhances the effectiveness of auditory feedback. In addition, we observed these significant differences despite using a between-subjects experimental design to compare established categories to new categories; it is likely that using a within-subjects design for testing subjects (with counter-balanced mixing of block order across subjects, for example) would reveal these effects even more strongly. Despite the design, within groups, individual participants showed similar trends as the group means: individuals in the NC and EC-A groups all had weaker performance than those in the EC-AT group.

The observation that these performance benefits were not shown for the established categories with without text group suggests that it is critical that the given vowel category is clearly identified for users. The auditory cues used in this study for the established categories with auditory cues group were provided using synthesized vowel feedback for one second. It is likely that most subjects did not successfully map these auditory target cues onto their underlying vowel categories from listening to these unnatural synthesized vowels. If listeners had been presented with a natural-speech example of the vowel target category, this would have been equivalent to the visual word cue example except for a slightly increased memory load (maintaining the naturally-spoken vowel word token in working memory instead of being able to read it on the screen *ad libitum*). Thus we expect that a task with a more natural auditory cue would yield the same performance as the visual cue performance observed here. Our results thus suggest it is necessary to provide users with an unambiguous representation of the target category in order to take advantage of categorical perception effects.

These results show that exploitation of human speech perception in auditory HMI design can drastically improve control. Development of more advanced auditory HMI designs is essential given the limitations of designs dependent on visual feedback: although these designs can be highly intuitive, they require intact vision from the user as well as a visual interface (external monitor) that must be kept in front of the users face and may thus interfere with natural communication. Although our results are promising, even greater improvements may be accomplished in the future by more precise mapping to participants' auditory perception. In the current study, all participants reported as native American English speakers and established categories targets were placed based on previously published vowel *production* data from speakers of American English. However, individual participants were likely to have individual variation in their own perceptual categories. An exaggerated example is differences due to regional dialects (e.g., northern Midwest vs. New England). We anticipate that future work to provide targets that are tuned to individual speaker perception and production will lead to further increases in performance.

### Motor control signals

The current study utilized sEMG as a motor control signal in order to capitalize on its high SNR and ease of use. Experiments using sEMG rather than more invasive and expensive technologies allow for a larger number of individuals to participate in HMI experiments. Our results about feedback modality performance are likely transferrable to other motor control signals. For instance, we found that participant performance on the task was close to that of a previous study, which made use of direct cortical recordings in a single locked-in patient to control real-time vowel synthesis [17]. Notably, this performance is achieved using sEMG signals instead of direct cortical recordings, suggesting that utilizing categorical vowel perception in providing a mode of feedback could yield excellent participant performance in a variety of HMI designs. In particular, recent work using electrocorticography (ECoG) has shown that recordings from the cortical speech network can be reliably used to control an HMI through overt (N = 2) and imagined (N = 2) phoneme articulation [25]. However, participants in this study relied on visual feedback for task completion. The constant attention to visual feedback required to control HMIs comprises a substantial cognitive load, whereas use of auditory feedback for HMI control has the benefit of potentially allowing simultaneous performance of visually-dependent tasks. Pairing the type of speech motor control scheme employed by Leuthardt et al. [25] with speech-related feedback such as the approach applied here to the EC-AT group has the potential to lead to improved performance and usability of HMIs. Future work is required to pair speech-related control signals to speech-related feedback, as well as to characterize the cognitive load associated with various types of HMI feedback schemes.

## Conclusions

Using sEMG, N = 24 healthy speakers of American English participated in 4 sessions over 3 days to learn to transition from visual and auditory (vowel synthesis) feedback to auditory feedback alone. A two-factor ANOVA showed a significant main effect of session and group (established categories with or without text cues and new categories), and a trend for an interaction between session and training group. These results suggest that by utilizing categorical vowel perception, users operating a HMI to produce continuous vowel sounds can achieve performance utilizing auditory feedback comparable to that achieved utilizing visual feedback. Moreover, the production of continuous vowel sounds generalizes beyond those explicitly trained in the HMI paradigm to other (untrained) vowel targets, but more so when natural (native English) vowel targets are used with unambiguously defined categorical targets.
